# A Qualitative Study to Compare Barriers to Improving Food Security among Households with Young Children in the U.S. as Perceived by Different Types of Stakeholders before and during COVID-19

**DOI:** 10.3390/nu15061438

**Published:** 2023-03-16

**Authors:** Elder Garcia Varela, Jamie Zeldman, Isabella Bolivar, Amy R. Mobley

**Affiliations:** Department of Health Education and Behavior, College of Health and Human Performance, University of Florida, Gainesville, FL 32611, USA

**Keywords:** food security, early childhood, community resources, health professionals, nutrition educators, nutrition policy, food assistance, COVID-19, systems integration

## Abstract

This qualitative study aimed to determine the perceived barriers of different community stakeholders’ to providing resources for improving food security in households with young children in the U.S. Community stakeholders working with low-income families with children 0–3 years of age in Florida were recruited to represent healthcare (*n* = 7), community/policy development (*n* = 6), emergency food assistance (*n* = 6), early childhood education (*n* = 7), and nutrition education (*n* = 6) sectors. In 2020, one-on-one interviews were conducted with each stakeholder in via Zoom, using an interview script based on the PRECEDE–PROCEED model and questions to capture the impacts of COVID-19. The interviews were audio-recorded, transcribed verbatim, and analyzed using a deductive thematic approach. A cross-tab qualitative analysis was used to compare data across categories of stakeholders. Healthcare professionals and nutrition educators indicated stigma, community/policy development stakeholders indicated a lack of time, emergency food assistance personnel indicated a limited access to food, and early childhood professionals indicated a lack of transportation as the main barriers to food security prior to COVID-19. COVID-19 impacts included the fear of virus exposure, new restrictions, lack of volunteers, and a lack of interest in virtual programming as barriers to food security. As perceived barriers may vary with respect to providing resources to improve food security in families with young children and the COVID-19 impacts persist, coordinated policy, systems, and environmental changes are needed.

## 1. Introduction

Food insecurity is a public health problem affecting millions of households with young children in the U.S [1]. In 2020, 15.3 percent (2.5 million) of U.S. households with children under the age of six reported being food insecure [1]. Food insecurity has been associated with poor diet quality [2,3,4,5,6], impacting the physical, cognitive, developmental, and social growth of young children [7,8,9,10,11]. Specifically, children under three who live in food-insecure households are more likely to be iron deficient, be at higher risk of cognitive–developmental problems, and experience more hospitalizations [12,13,14].

The World Health Organization declared COVID-19 a global pandemic in March 2020 [15], impacting many households, specifically those living in low-income communities [16,17,18]. Unemployment rates related to COVID-19 resulted in individuals reporting difficulty in obtaining healthy and affordable foods for themselves and their children [18,19,20,21], exacerbating existing racial/ethnic and socioeconomic inequities [22,23].

While the existing literature provides evidence of an association between food insecurity and adverse health outcomes in young children [14,24,25,26,27,28], minimal research has been conducted to address the barriers to food security in households with children under three [18]. The research suggests community stakeholders offer a unique perspective regarding community needs, barriers, and opportunities [29]. For this study, community stakeholders included key individuals, groups, and/or organizations who share a vested interest in a specific topic or subpopulation, such as food security in households with young children. Thus, engaging community stakeholders in the research process is beneficial to (1) obtain a better understanding of the community’s needs and priorities; (2) increase community buy-in in the proposed program; and (3) develop a sense of shared responsibility for community health [29,30]. Research also shows that the meaningful and equitable engagement of multiple stakeholder groups can contribute to developing and implementing better quality, more acceptable, and relevant health programs, policies, and services [31,32]. By bringing different perspectives, these stakeholders provide valuable input on the processes, outcomes, and lessons learned from their niche that can contribute to reducing existing health disparities [31,32]. Nonetheless, little is known about how perceived barriers may vary among different types of stakeholders with respect to improving food security in households with young children.

The objective of this study was to explore the perceptions of different types of community stakeholders (i.e., healthcare providers, early childhood education specialists, community health planning and policy development professionals, emergency food assistance providers, and nutrition education professionals) regarding the barriers to providing and delivering services and resources to individuals with children under three years of age who experienced food insecurity before and during COVID-19. Identifying the factors that impact access to services and resources for improving food security and how these perceived barriers may vary by the type of stakeholder is a critical step in adapting existing programs and policies to improve the health and quality of life of food-insecure households with young children.

## 2. Materials and Methods

Qualitative, in-depth interviews were conducted with community stakeholders in Florida. The Consolidated Criteria for Reporting Qualitative Research (COREQ) guidelines (Supplementary File S1), a 32-item checklist for interviews and focus groups, guided the reporting of study findings.

### 2.1. Study Participants and Sampling

Purposive sampling was supplemented with convenience sampling to recruit participants for this study [33]. Potential participants included community stakeholders with a vested interest in supporting families with young children experiencing food insecurity. We defined community stakeholders as individuals working in local groups, organizations, and businesses who directly provide services and/or resources to improve the food security status of families with children under three years of age. Multiple stakeholders throughout the state of Florida were identified from publicly available information online (i.e., websites, reports, etc.) in various sectors, including healthcare, community health planning and policy development, emergency food assistance, early childhood education, and nutrition education. Participants were recruited via email and phone by a member of the research team (E.G.V.) to seek their participation with the goal of including somewhat equal representation from various categories or types of stakeholders (i.e., healthcare providers, early childhood education specialists, community health planning and policy development professionals, emergency food assistance providers, and nutrition education professionals). Flyers were included in electronic communication to be shared with individuals within the targeted organizations. This paper does not report the stakeholders’ employers and job titles to retain the anonymity of the participants. After completing the in-depth interview (response rate of 85%), stakeholders who participated in this study received monetary compensation through a $30 electronic gift card.

Table 1 illustrates participants’ characteristics by stakeholder type. A total of 32 community stakeholders participated in semi-structured interviews, including healthcare providers (*n* = 8), early childhood education specialists (*n* = 7), community health planning and policy development professionals (*n* = 6), emergency food assistance providers (*n* = 6), and nutrition education professionals (*n* = 5). Overall, the majority of stakeholders interviewed were non-Hispanic (91%), white (64%) females with at least a Bachelor’s degree education level (84%) and an average of 8 years of experience providing services and resources to individuals with children under the age of three years.

### 2.2. Data Collection

Data were collected through in-depth, semi-structured interviews via Zoom. The interviews lasted approximately 90 min and were conducted between November 2019 and August 2021 by one of the study members (E.G.V.). The semi-structured approach included follow-up questions for further exploration of the topic. The interview guide was developed based on the Predisposing, Reinforcing, and Enabling Constructs in the Educational Diagnosis and Evaluation (PRECEDE) component of the PRECEDE–PROCEED model (Appendix A). The PRECEDE–PROCEED model provides a comprehensive approach to addressing individuals’ health and quality of life by assessing the needs for designing, implementing, and evaluating health-promotion programs [34]. Thus, this study utilized this model to inform the development of a community-based intervention that addresses the needs of individuals with young children who are experiencing food and nutrition insecurity. The interviews were audio recorded. Permission to record was obtained from the participants via a waiver of documentation of informed consent and verbally at the beginning of the interview. The interviews were transcribed verbatim via Zoom and cross-checked by a study member for accuracy. The interviews were conducted until data saturation was reached, which was guided by the seven parameters identified by Hennink et al. 2017 [35]. A brief demographic survey was also administered via Qualtrics (Appendix B) to summarize the participant characteristics.

### 2.3. Data Analysis

The data analysis was conducted by three female researchers, E.G.V., J.Z., and I.B., with 80% of the transcripts being independently double-coded and compared for consistency [36]. For the double-coded interviews, disagreements between researchers were resolved through discussion [37]. For the single-coded interviews, any uncertainty in coding by one researcher was discussed with the other two researchers before categorization. Specifically, data were analyzed via traditional text analysis using a deductive thematic approach [37]. As such, the PRECEDE component of the PRECEDE–PROCEED model was conceptually used to identify the themes and subthemes. A qualitative cross-tabulation analysis compared pre- and existing COVID-19 data among the different types of stakeholders, organized into five categories (i.e., healthcare, community health planning and policy development, emergency food assistance, early childhood development, and nutrition education). For this paper, the following questions were selected for qualitative analysis: (1) What were the main barriers to providing or delivering services or resources to these families prior to COVID-19? (2) What were the main barriers in providing or delivering services or resources services or resources to these families during the COVID-19 response? (3) What resources are lacking/not available or are needed, especially for families with infants and toddlers ages 0–3 years old? Findings from the other questions were analyzed and presented in a previously published manuscript [38]. The demographic questionnaire was analyzed using IBM SPSS Statistics 29.

## 3. Results

### 3.1. Thematic Analysis Results

The results were organized according to Phase 4: Educational and Ecological Assessment of the PRECEDE–PROCEED model and compared among the different types of stakeholders. Table 2 illustrates the predisposing, reinforcing, and enabling factors that impact stakeholders across different sectors to provide and/or deliver services and/or resources to this population.

#### 3.1.1. Barriers to Providing and/or Delivering Services and/or Resources to Improve Food Security among Households with Children under Three before COVID-19

*Lack of or limited access to transportation (enabling factor).* Early childhood education specialists suggested that a lack of or limited access to transportation was their biggest challenge for program participation. One early childhood education specialist stated, “I think one of the big [issues], I would say, is transportation for the parent and child to get to the activity or to where we’re having the program” (P4). Although a lack of or limited access to transportation was not the most salient barrier for other stakeholders (i.e., healthcare providers, emergency food assistance providers, and nutrition education professionals), they also mentioned that transportation was a challenge for their clients in receiving services and/or resources. In contrast, community health planning and policy development professionals did not mention a lack of or limited access to transportation as a barrier.

*Logistical issues with recruiting and retaining participants (reinforcing factor).* Stakeholders also mentioned logistical issues (i.e., scheduling, length of visit, cost, etc.) to recruiting and retaining participants as some of the most frequent challenges across sectors (i.e., healthcare, early childhood education, community health planning and policy development, and nutrition education). One participant mentioned, “the time that it takes to go into the office, the number of office visits, you know the frequencies up to four times a year and more depending on if you have multiple children and if their appointments are not aligned… it is a challenge for them to receive assistance” (P17). While this was the second-highest-mentioned barrier across stakeholder types, it was not the top mention for any of the identified groups. Further, emergency food assistance providers did not mention logistical issues with recruiting and retaining participants as barriers to providing and/or delivering services to their clients.

*Pride and stigma (predisposing factor).* Stakeholders suggested pride and the stigma associated with accessing resources and/or services were a challenge to supporting individuals with young children. Community health planning and policy development, healthcare, and nutrition education stakeholders suggested pride and stigma were issues in providing and delivering services to the target population. Specifically, healthcare providers indicated pride and stigma were two of the most challenging barriers to providing services and resources to households with young children. One participant mentioned, “There’s a lot of pride. They really don’t want to rely on services. They don’t want to be looking for a handout. It’s really trying to break down that barrier and let them know that we’re not judging them” (P2).

While a lack of or limited access to transportation, logistical issues with recruiting and retaining participants, and pride and stigma were the most frequently mentioned barriers across the different types of stakeholders, each group also expressed its unique challenges. For instance, community health planning and policy development stakeholders noted that individuals’ lack of time for seeking out or receiving services and resources was their biggest challenge to providing service and resources before COVID-19. Further, nutrition education professionals mentioned an inequality of resources across neighborhoods as the most salient barrier when recommending services and/or resources to their program participants.

#### 3.1.2. Barriers to Providing and/or Delivering Services and/or Resources to Improve Food Security among Households with Children under Three during COVID-19

*COVID-19 restrictions (enabling factor).* Overall, stakeholders across all sectors (i.e., healthcare, community health planning and policy development, early childhood education, emergency food assistance, and nutrition education) suggested that COVID-19 restrictions (i.e., social distancing, self-isolation, shutdowns, curfews, etc.) were some of the main barriers to providing and/or delivering services and/or resources during COVID-19. Specifically, community health planning and policy development stakeholders mentioned that COVID-19 restrictions prevented them from providing their services during regular hours. One participant stated, “We’re only open from nine to one… and then sometimes, you know, we can only take appointments every 10 to every 15 min. So, we usually take between 18 to 20 appointments per day. That is a real challenge” (P9).

*Virtual programming limitations (enabling and predisposing factor)*. Stakeholders mentioned that because of COVID-19 restrictions, they had to adjust their programming to reach participants in different forms, including through online platforms. However, virtual programming brought challenges to providing or delivering services and resources. While virtual programming challenges (i.e., consent, face-to-face preference, etc.) did not affect all stakeholders, early childhood education professionals, healthcare providers, and nutrition education professionals stated that not having the flexibility to meet in person with their clients restricted their ability to access the population they served. Additionally, limited access to technology and internet connectivity made it particularly challenging for individuals to stay connected and consistently engage in programming. One stakeholder mentioned, “Everybody doesn’t necessarily have a computer in their home or really reliable Internet service. So, while I want to believe, we were still reaching a good number of people, I mean, we do have to face the reality that we were not probably reaching nearly as many people as we were before simply because, you know, just lack of technology, not comfortable with it, not savvy with it. Also, I would imagine, in the grand scheme of things, when people were trying to pay bills and, at the height of this, you know, keeping the Internet on might not have been the top priority” (P29).

*Fear of COVID-19 exposure (predisposing factor).* Stakeholders also mentioned that the fear of COVID-19 exposure was one of the most salient challenges when providing or delivering services and/or resources to households with young children during COVID-19. Healthcare providers mentioned that families were not making their scheduled appointments primarily due to the fear of being exposed to the virus. One stakeholder stated, “A lot of families didn’t feel comfortable coming to the doctor. You know, understanding that a doctor’s office might be a higher chance of getting COVID from other patients. So, we had a lot of concerns, and we’re correcting for it now, but there’s still a backlog of a lot of families that did not seek routine care, so their basic needs were not identified. And now, I’m worried that we will end up missing a lot of families that we sort of would have seen more routinely and have screened for before the pandemic through basic primary care” (P23). Nevertheless, not all types of stakeholders (i.e., emergency food assistance and early childhood education) identified a fear of COVID-19 exposure as a barrier to providing or delivering services.

*Lack or limited number of volunteers (reinforcing factor).* Stakeholders also mentioned the lack of or limited number of volunteers was a barrier to providing or delivering resources and services to individuals with young children. Specifically, emergency food assistance and healthcare providers mentioned this barrier as their most salient challenge in providing services and/or resources. One stakeholder mentioned, “Our volunteers are aging out because we’re a 34-year-old pantry, and so they’re aging out. And a lot of them are afraid to come around, you know, other people, so we’ve been really struggling providing volunteers, younger volunteers, healthy volunteers than we had before” (P15). Similar to the fear of COVID-19 exposure, not all types of stakeholders identified the lack or limited number of volunteers as their number one barrier to providing or delivering services in the community.

While COVID-19 restrictions, virtual programming limitations, fear of COVID-19 exposure, and lack of or a limited number of volunteers were the most salient barriers across all types of stakeholders during the COVID-19 pandemic, stakeholders across sectors also recognized that the barriers identified before COVID-19 were exacerbated during the pandemic. For instance, one participant mentioned, “At the height of the pandemic, accessing food was difficult because of transportation. You know, limited availability of public transport” (P25). Additionally, stakeholders mentioned that COVID-19 restrictions disrupted their employment status due to the lack of or limited accessibility to childcare options during the pandemic.

#### 3.1.3. Services and Resources Needed among Households with Children under Three during the COVID-19 Pandemic

*Access to affordable and high-quality childcare services*. Overall, most stakeholders across the different sectors indicated that families with children under the age of three needed access to affordable and quality childcare services. Healthcare providers specifically stated that affordable childcare services are inadequate and prevent mothers from continuing to work and support their families. For example, one participant said, “Quality daycare and preschool is probably number one for that age group because if parents have that, then, the kids, you know, the kids would be well cared for, and the parents could be able to then go to school, find jobs, be able to do what they need to do to support their family” (P27).

*Marketing strategies to increase awareness about available services and resources.* Stakeholders suggested that better communication and marketing strategies are needed to raise individuals’ awareness about the available services and resources in their communities. Specifically, nutrition education and community health planning and policy development professionals mentioned marketing strategies as the most-needed strategy. One participant stated, “There’s a lack of overall knowledge. So even if sometimes the resource is available, they may not know about it because you know no one’s ever told them, or they just don’t know where to find it. Better communication or marketing strategies are one of the main things a lot of the health services are lacking out there” (P32).

*Centralized referral system.* Stakeholders across the different sectors (i.e., healthcare, early childhood education, emergency food assistance, and nutrition education) also suggested the need for a centralized referral system so that individuals are evaluated, efficiently matched, and directed to the programs and/or services that address their needs. One emergency food assistance provider mentioned, “I think continuity of service is extremely important, and that technology piece that we share in terms of a direct flow referral process is [important]. For example, if a client goes to seek assistance at a baby-friendly pantry and they’re able to offer access to a diaper pantry, it would be great to have a direct referral system where they’re able to then refer the client to us. We’re able to close that loop for food assistance because we know folks are seeking assistance…one economic trade-off, then they are likely in need of another.”

## 4. Discussion

Understanding the specific needs and barriers faced by different types of community stakeholders can inform the development and adaptation of programs and/or strategies that better fit the needs of those implementing the program and those receiving the services and resources [18,39,40]. As the previous research suggested, a multi-sector response is essential to coordinating community support and increasing the access to unmet social needs, specifically with respect to addressing food security [16,41,42,43]. Moreover, exploring the barriers and needs of different types of stakeholders during times of economic, political, or social crises (i.e., the COVID-19 pandemic) is critical for identifying the short- and long-term implications of the infrastructure that impacts the development and implementation of policies and programs intended to improve the health and quality of life of individuals in these communities [16,18,44].

Specifically, the COVID-19 pandemic exacerbated existing barriers (i.e., a lack of or limited transportation, pride, stigma, etc.) to providing services and resources to individuals with young children. It also generated a new set of challenges with additional implications. For instance, COVID-19-related restrictions, such as social distancing, limited hours of operation, and shutdowns, impacted all community sectors (i.e., healthcare, early childhood education, community health planning and policy development, emergency food assistance, and nutrition education). However, healthcare providers, stated that individuals were particularly reluctant to bring their children in for routine check-ups due to the potential risk of exposure to COVID-19. Recent studies have identified similar findings, suggesting that caregivers missed routine pediatric care due to the fear of contracting COVID-19, increasing the risk of morbidity and mortality associated with treatable and preventable health conditions [45,46].

Despite the efforts to transition to virtual programming amidst the COVID-19 outbreak, stakeholders faced many challenges in reaching participants and providing high-quality services to their clients. Early childhood education professionals, healthcare providers, and nutrition education professionals experienced many challenges in staying connected and engaging with individuals with young children. Recent studies have identified similar findings. suggesting that programs that adapted their delivery of services to a virtual platform during the COVID-19 pandemic were able to support individuals who did not feel comfortable receiving in-person services and resources [47,48]. However, similar studies also suggested that these innovative strategies further exposed existing inequalities in low-income communities, including a lack of technology and internet access [47,49,50].

While stakeholders across groups shared similar perspectives regarding the barriers to providing and/or delivering services and/or resources, each type of stakeholder group experienced different barriers before and during COVID-19. For instance, none of the stakeholder groups suggested the same barrier as their main (i.e., most frequently mentioned) barrier when discussing challenges to providing services and resources before COVID-19. Similarly, none of the stakeholder groups reported the same barrier as their top barrier before and during COVID-19. These findings provide additional evidence of the importance of involving key stakeholders to inform the development and implementation of health promotion programs and policies [31,51]. Community program implementers play an essential role, especially in identifying the assets and resources available to meet the needs of the populations they serve, in helping to identify the needs, in informing best practices, and in adapting health promotion programming [31,51,52].

Overall, stakeholders (i.e., healthcare providers, early childhood education professionals, community health planning and policy development specialists, and nutrition education professionals) expressed the need to improve the knowledge and awareness of individuals about the programs and resources available to them to reduce food insecurity. The research has identified similar findings, suggesting a lack of community outreach opportunities and marketing strategies to improve individuals’ awareness about community resources and assistance programs [53,54,55,56]. Furthermore, stakeholders (i.e., healthcare providers, early childhood education professionals, emergency food assistance providers, and nutrition education professionals) also addressed the need for a centralized referral system to better address the needs of community members. A recent study found that linking clinical services to community-based resources is a promising strategy for assisting individuals with chronic disease prevention and management [57]. Finally, stakeholders (i.e., healthcare providers, early childhood education professionals, community health planning and policy development specialists, and nutrition education professionals) also expressed the need for affordable, high-quality childcare services for their clients. When compared with all the other types of stakeholder groups, community health planning and policy development specialists did not share many suggestions for improving the food security of the target population. Nevertheless, they highlighted the importance of affordable healthcare services and resources to improve the well-being of these individuals and their children.

### Limitations

While this study offers an overview of the unique barriers faced by various types of community stakeholders when providing services to individuals with young children experiencing food insecurity, these findings may not be transferable to other settings and populations. Additionally, given that a purposive sampling method was supplemented by convenience sampling to recruit participants for this study and that most participants were female, the participants’ responses may not represent the perspectives of all stakeholders working or providing services within the selected sectors. Likewise, the views of the community stakeholders may reflect the needs of the communities in Florida in which they provide services. Moreover, although the overall number of participants (*n* = 32) was adequate for a qualitative study, the number of recruited participants per type of community stakeholder group was modest and may not be reflective of the perceptions of all stakeholders that might identify themselves as part of these groups. Lastly, because interviews were conducted during or a few months after the onset of the COVID-19 pandemic, participants’ responses may represent their present-day experiences. While rigorous data collection techniques were employed to minimize potential bias, future research should explore the perceptions of caregivers of young children as they may indicate different barriers or rank the identified barriers in a different order.

## 5. Conclusions

Despite the current availability and implementation of existing programs and policies to improve food security in the U.S., various barriers continue to prevent the provision of services or resources by community stakeholders to households with young children. Additionally, these barriers were further exacerbated or new barriers emerged as a result of COVID-19. Interestingly, these barriers often varied across stakeholders, indicating a potential need to centralize the delivery or availability of food security and related services and resources for families with young children.

Understanding the existing gaps and potential opportunities within each sector is important for supporting adapting programs and policies, especially after experiencing the impact of COVID-19. Future research should focus on evaluating collaborative or centralized strategies across different types of stakeholders with respect to improving food security in vulnerable populations, including households with children under the age of three.

## Figures and Tables

**Table 1:** Demographic characteristics by community stakeholder type.

Demographic Characteristics	Overall (*n* = 32)	Healthcare (*n* = 8)	Early Childhood Education (*n* = 7)	Community Health Planning and Policy Development (*n* = 6)	Emergency Food Assistance (*n* = 6)	Nutrition Education (*n* = 5)
Age, mean (SD)	42 (11.4)	45 (8.2)	50 (10.4)	37 (9.7)	39 (15.0)	36 (9.5)
Gender, % (*n*)						
Male	6	13	0	0	0	20
Female	94	88	100	100	100	80
Race, %						
Asian	6	13	0	0	0	20
Black or African American	22	0	71	0	50	40
White	63	88	29	100	50	40
Other	9	0	0	0	0	0
Ethnicity, non-Hispanic, %	91	100	100	83	67	100
Highest level of education, %						
Some college or technical school	13	0	29	0	33	0
Associate degree	3	0	14	0	0	0
Baccalaureate degree or higher	84	100	57	100	67	100
Years of work experience, mean (SD)	8 (7.8)	13 (10.2)	9 (6.5)	2 (2.3)	7 (6.5)	8 (8.9)

**Table 2:** Barriers to providing and/or delivering services and/or resources to improve food security among households with children under three before and during the COVID-19 pandemic.

Type of Stakeholder	Before COVID-19	Quotes	During COVID-19	Quotes
Healthcare providers (*n* = 8)	Pride and stigma (top barrier)	“Families not wanting to necessarily be completely open about how insecure they might be when it comes to food because I do feel like when I try to ask more questions to see what kind of resources families might need, they usually don’t give a whole lot of detail, and tend to sort of say that everything is okay. So, I think families probably don’t want to seem like they don’t have enough for their kids when they’re seeing me for a visit” (P24)	Fear of COVID-19 exposure (top barrier)	“There’s still a backlog of a lot of families that that did not seek routine care, so their basic needs were not identified. I’m worried that we ended up missing a lot of families that we sort of would more routinely have screened for before the pandemic through basic primary care. I’m very worried that through the lack of our normal preventive care services that acute problems like food insecurity will be missed” (P23)
	Logistical issues with recruiting and retaining participants	“Most of them are on Medicaid or some kind of public assistance. So they have coverage for their kids. It is more being able to reach them to schedule appointments. They may not have phones or internet service” (P27)	COVID-19 restrictions/safety protocols	“Initially when everything shut down, families just… they weren’t coming to the clinic at all. So, their kids weren’t getting any care. We tried… we developed a protocol of what to do if your kid was sick and how to contact us so that if they needed to come in for an emergency that would happen” (P27)
	Lack of or limited access to transportation	“I see possibly transportation barriers to obtaining food or to get to a medical center” (P16)	Lack of or limited number of volunteers	“One of the barriers that got worse was the manpower distribution. So, having enough volunteers around the pregnancy center to work with the moms, meet with the moms. meet with the children, assess the need, and get those resources distributed” (P21)
Early childhood education (*n* = 7)	Lack of or limited access to transportation (top barrier)	“The only thing that really came across as an issue was transportation if we wanted to provide resources at a central location. At first, we had partnerships with churches… they were able to transport everyone in the program. Then, as time went on budget, it was modified, and we were only able to transport those that we’re currently pregnant. Then it turned to those that were high risk pregnancy, and so on.” (P26)	Virtual programming limitations (top barrier)	“I think one of the barriers that we have come across is when we first shut down…just like now we’re doing phone visits. And some of the families, even though we were doing home visits, some of the families did not want… we couldn’t even do like facetime because they thought there was a HIPPA thing, even though we were going into the homes” (P12)
	Lack of participant motivation/interest to engage in community programs	“It’s getting parents engaged in these activities and have them take advantage of the services that we are providing.” (P4)	COVID-19 restrictions/safety protocols	“Definitely not been able to be in person with families. We were kind of lost because of COVID. Not providing that in person, hands on you know direct service, where we can bring things to them at the church where they can come and benefit from it.” (P26)
	Logistical issues with recruiting and retaining participants	“If parents don’t have like a person to help them get the child… pick their child up from school because their (job) hours are from eight to three. They are unable to receive services” (P3)	Lack of or limited access to technology/internet	“Some of us, like me myself, not really having computers… Or you might have one and then maybe the parents don’t have that access to you know the Internet as well, so that can be a barrier in it within itself too” (P12)
Community health planning and policy development (*n* = 6)	Participants’ lack of time to seek out or receive services/resources (top barrier)	“The time that it takes to go into the office and the travel that it takes to go into the office, the number of office visits, you know the frequencies up to four times a year and more depending on the if the if you have multiple children and if their appointments are not aligned” (P17)	COVID-19 restrictions/safety protocols (top barrier)	“We’re only open from nine to one. And then sometimes we can only take appointments every 10 to 15 min. So, we usually take between 18 to 20 appointments per day” (P9)
	Pride and stigma	“Finding ways for food programs to be culturally appropriate, accessible based on date, time and location and in an appropriate way so that the stigma is reduced as much as possible” (P14)	Exacerbation of existing barriers	“COVID 19 has really exacerbated all of the barriers: time, physical presence, ease of use, but, in some ways, those- some barriers have been eased or alleviated through waivers to the programs” (P17)
	Logistical issues with recruiting and retaining participants	“Some of the challenges are at the provider level. So, the amount of paperwork that providers have to do to be able to provide CACFP- reimbursed meals through CACFP. There’s a lot of paperwork that providers have to go through” (P17)	Lack of available resources	“A lot of parents couldn’t come to pick up food boxes for up to 3 weeks. We didn’t have the manpower like you know, we had a family of four that had 12 boxes or 16 boxes of food. We don’t have the capabilities… we don’t have a truck or anything like that where we load up the food and then provided to them that way” (P19)
Emergency food assistance (*n* = 6)	Lack of or limited access to and availability of services and resources (top barrier)	“I think that the lack of resources, meaning that our pantries, not always, they’re not always full. Meaning we need more resources to meet the needs of the community. So, one of the biggest barriers is that we may run out a formula in the middle of the month. And we are not able to get them until a week later, two weeks later, maybe the following month” (P1)	Lack of or limited number of volunteers (top barrier)	“We need more volunteers. Our volunteers are aging out because we’re a 34-year-old pantry and so they’re aging out. And a lot of them are afraid to come around you know other people, so we we’ve been really struggling providing volunteers, younger volunteers, healthy volunteers than we had before” (P15)
	Lack of or limited number of volunteers	“Having those consistent volunteers to rely on and setting that up within those communities that need it the most. So, that sometimes is a little bit of a struggle, is just establishing those additional community connections through our partner agencies that we utilize” (P10)	Lack of funding to support access to services and resources	“This year, many fundraising events didn’t take place because she couldn’t go be out there. So, the lack of funding resources easily available to intern, get this, some needed items is also a challenge” (P1)
	Lack of funding to support services and resources	“Most of our funding is through grants and federal funding, state funding, and things of that nature. Making sure that we have enough money for the salaries, building maintenance, and then being able to look at the budget and other resources to possibly extend it” (P8)	Lack of centralized outreach platform to promote resources and services	“Data sharing amongst social services. A case management platform to be able to better understand the folks that we serve, and as we transition our agencies to also utilize this platform. We find it challenging to support an adequate referral process” (P18)
Nutrition education (*n* = 5)	Lack of or limited access to transportation (top barrier)	“We deal with the low-income population and some of our families stay in rural areas where it takes them about 10 to 15 min to get to grocery store. They don’t have car, so they are relying on basically the convenience stores” (P30)	Virtual programming limitations (top barrier)	“Sometimes being able to communicate with them over the phone if there are Spanish speaking client because they don’t they don’t fully they don’t fully understand what sometimes what we’re saying when we’re when we’re talking to them over the phone versus like being in the office” (P30)
	Logistical issues with recruiting and retaining participants	“When it comes to capturing the providers, the caretakers…there’s always competing things you know, whether it’s jobs, time off, just not having the time in the day, transportation, etc.” (P29)	COVID-19 restrictions/safety protocols	“When we kind of started getting pushed out of our offices and out of the sites was right around the time when schools were having breaks anyways for spring break. So it was kind of happening more for the adults. But once we got pushed out of our offices and kind of ceased, it was, it was difficult. I’m not gonna lie.” (P29)
	Pride and stigma	“There’s a lot of pride and they really don’t want to rely on services. They don’t want to be looking for a handout. So, it’s really trying to break down that barrier to and let them know that we’re not judging them” (P2)	Fear of COVID-19 exposure	“People will feel like they have to come in to get services, but at the same time they’re also concerned, or they’re scared that if they come in, they can come in contact with someone who has COVID so that’s like kind of a barrier because people will skip out on appointments.” (P32)

## Data Availability

Not applicable.

## Supplementary Materials

The following supporting information can be downloaded at: https://www.mdpi.com/article/10.3390/nu15061438/s1, File S1: COREQ (COnsolidated criteria for REporting Qualitative research) Checklist [58].

## Appendix A. Interview Moderator Guide

Good morning/afternoon. Thank you for agreeing to participate in this study. My name is_____. I will be conducting the interview today. We also have another study member, _____, on the line. They will be helping to take notes.

This study explores community stakeholders’ perceptions regarding the challenges and opportunities to obtaining or providing adequate, high-quality, and age-appropriate food for children 0–3 years old in low-income communities before and as a response to COVID-19. We ask you to keep the video feature off for the interview duration. We will only be recording audio from this interview. This interview will take approximately 60–90 min.

Participation may help you identify ways to improve your current practices to provide adequate, high-quality, and age-appropriate food for infants and toddlers. Participation is voluntary. There are no costs or likely risks associated with participation. You may discontinue participation at any time. If you complete the session, you will receive a $30 incentive in the form of an Amazon gift card, which will be emailed to the email you provided within 24–48 h.

Do you have any questions before we get started?

*Start Recorder*

1. Please describe the services or resources you/your program/organization provides to families with infants and toddlers 0–3 years old.
  - What services or resources does your program/organization offer to address food security?
  Note: If the participant does not understand the term food security, read the USDA definition below: “Food security for a household means access by all members at all times to enough food for an active, healthy life.
2. How much of a concern was food security among the families you serve prior to COVID-19?
3. How much concern has food security among families been during the COVID-19 pandemic?
4. What were the main barriers to providing or delivering services or resources to these families before COVID-19?
  - Social (e.g., lack of community support, social norms)
  - Economic (funding/donations)
  - Policy (regulations)
5. What were the main barriers to providing or delivering services or resources services or resources to these families during the COVID-19 response?
  - Social (e.g., lack of community support, social norms)
  - Economic (funding/donations)
  - Policy (regulations)
6. What do you think were the major barriers to food security for local families with infants and toddlers ages 0–3 years prior to COVID-19? (Note: Overall opinions about food insecurity)
  - Lack of access/transportation to program offices or adequate foods
  - Lack of awareness/eligibility for federal assistance.
  Poverty-related issues (housing, health care, substance use, unemployment, utilities)
7. What do you think were the major barriers to food security for local families with infants and toddlers ages 0–3 years during the COVID-19 response? (Overall opinions about food insecurity)
  - Lack of access/transportation to program offices or adequate foods
  - Lack of awareness/eligibility for federal assistance/stigma associated with applying for federal assistance
  - Poverty-related issues (housing, health care, substance use, unemployment, utilities)
  - Lack of childcare support/options
8. What foods are most challenging for parents to obtain for infants and toddlers ages 0–3 years as a result of COVID-19?
  - What would make it easier for parents to obtain these foods?
9. What resources are available in the community to address these barriers prior to COVID-19?
  - How do parents learn about the resources available in the community?
10. What resources are available in the community to address these barriers due to COVID-19?
  - How do parents learn about the resources available in the community?
11. In your opinion, what resources are lacking/not available or are needed, especially for families with infants and toddlers ages 0–3 years old?
  - What resources would you like to see to improve food security for families with infants and toddlers 0–3 years old?
12. Is there anything else you would like to share regarding food security for infants and toddlers ages 0–3 years old?
  *Stop Recorder*

## Appendix B. Demographics Survey

Please tell us about yourself:

1. What is your gender?
  - Male (1)
  - Female (2)
2. What race do you consider yourself to be?
  - White (1)
  - Black or African American (2)
  - American Indian or Alaska Native (3)
  - Native Hawaiian or other Pacific Islander (4)
  - Asian (5)
  - Other (please explain) (6): __________________________________________
3. Do you consider yourself to be Hispanic or Latino?
  - No (1)
  - Yes (2)
4. What is your age? _________ Years
5. What is the highest level of education you received?
  - Less than high school (1)
  - High school diploma or GED (2)
  - Some college or Technical school (3)
  - Associate’s degree (4)
  - Bachelor’s degree or more (5)
6. How long have you been working in your current position (years)? ____________
7. What is the zip code of your work location? ______________
